# Reviewed article: Martins CI, Silva CSO, Souza FG, Faria SMC, Silva LR, Camargos MCS. Sociodemographic profile of elderly people cared for at a medium and high complexity hospital in Belo Horizonte, Minas Gerais, Brazil: a cross-sectional study. Epidemiol Serv Saude. 2025:34;e20240254

**DOI:** 10.1590/S2237-96222025v34e20240254.b

**Published:** 2025-09-08

**Authors:** Luiz Milton Gorzoni

**Affiliations:** Faculdade de Ciências Médicas da Santa Casa de São Paulo, Departamento de Clínica Médica

**Table:** 

Rating	Excellent	Good	Average	Below average	Poor
Interest				✓	
Quality				✓	
Originality				✓	
Overall				✓	

**Table:** 

Questionnaire	Yes	No	Not applicable
Does the manuscript contain new and significant information to justify publication?		✓	
Does the Abstract (Summary) clearly and accurately describe the content of the article?	✓		
Is the problem significant and concisely stated?	✓		
Are the methods described comprehensively?	✓		
Are the interpretations and conclusions justified by the results?	✓		
Is adequate reference made to other work in the field?	✓		

## Manuscript Structure

Length of article is: Adequate

Number of tables is: Adequate

Number of figures is: Adequate

Please state any conflict(s) of interest that you have in relation to the review of this paper (state “none” if this is not applicable): None

## Comments

The objective of this manuscript is very interest but it is necessary to review de Portuguese redaction style.

Its data are very basic. It is possible that there are more data to show and discuss.

